# Multi-scale Fusion of Stretched Infrared and Visible Images

**DOI:** 10.3390/s22176660

**Published:** 2022-09-02

**Authors:** Weibin Jia, Zhihuan Song, Zhengguo Li

**Affiliations:** 1 School of Aeronautics and Astronautics, Zhejiang University, Hangzhou 310027, China; 2 School of Control Science and Engineering, Zhejiang University, Hangzhou 310027, China; 3 SRO Department of Institute for Infocomm Research, Singapore 138632, Singapore

**Keywords:** multi-scale fusion, infrared image, visible image, content-adaptive gamma correction, weight measures

## Abstract

Infrared (IR) band sensors can capture digital images under challenging conditions, such as haze, smoke, and fog, while visible (VIS) band sensors seize abundant texture information. It is desired to fuse IR and VIS images to generate a more informative image. In this paper, a novel multi-scale IR and VIS images fusion algorithm is proposed to integrate information from both the images into the fused image and preserve the color of the VIS image. A content-adaptive gamma correction is first introduced to stretch the IR images by using one of the simplest edge-preserving filters, which alleviates excessive luminance shifts and color distortions in the fused images. New contrast and exposedness measures are then introduced for the stretched IR and VIS images to achieve weight matrices that are more in line with their characteristics. The IR and luminance components of the VIS image in grayscale or RGB space are fused by using the Gaussian and Laplacian pyramids. The RGB components of the VIS image are finally expanded to generate the fused image if necessary. Comparisons experimentally demonstrate the effectiveness of the proposed algorithm to 10 different state-of-the-art fusion algorithms in terms of computational cost and quality of the fused images.

## 1. Introduction

Image fusion is acknowledged as a significant manipulation in surveillance, advanced military, and object perception [1,2,3]. In addition, fusion has also been used for high dynamic range imaging [4], dehazing [5], low-light image brightening [6], and high-resolution depth maps imaging [7]. The fused image combines complementary sources information from multiple images of various sensors that contains better scene details than the original one [8,9]. In multi-sensor image fusion, a popular topic is to consolidate the infrared (IR) and visible (VIS) images.

The VIS and IR images are one couple of complementary data [10,11]. The VIS images are captured by optical sensors, which are high in contrast and have considerable details. The IR band sensors capture thermal emanation coming out from the various objects, making it easy to identify the prominent targets from the complicated environment. The hidden properties of the scenes may not be visible to the unaided eyes [12]. The IR images are seldom disturbed by dark situations, low-light conditions, concealed information, and fog [13]. Because the objects are detected based on the amount of heat radiation, the brightness of humans will be higher in a low-temperature environment at night, which is also one of the concerns of the IR images. The IR band sensors can capture images with enhanced visibility of scenes dropped in the VIS images. The IR band sensors aid the human visual system in gathering effective information from the scenes.

On the other hand, the IR band sensors also have some limitations. The perception of the IR band sensors is material-dependent, and the IR images are in grayspace. Objects made from the same material but having different colors are indistinguishable concerning the IR band sensors [14,15]. Consequently, the objects made from the same material are perceived texture-less in the IR images [8]. Therefore, it is important to fuse the IR and VIS images. Due to the advantages of the IR images, the application fields of the IR band sensors are being used in an increasingly wide range of scenes. For example, to better apply the exploration performance of the IR band sensors and solve the problem of low exploration efficiency of the radar in some cases, the new coatings of facilities materials are being developed to increase the reflection of the IR band. Therefore, even though the same methodology can be adopted for the VIS band, humans will not feel comfortable due to the stronger reflection. It is desired to fuse the IR and VIS images, such that the fused image also benefits from the new coatings.

In the past few decades, dozens of pixel-level image fusion methods that directly integrate information on corresponding pixels from two or more images were proposed to combine the VIS and IR images [16]. Typical examples are wavelets, multi-resolution singular value decomposition (MSVD), and curvelet transform (CVT) [17,18,19]. The problem of determining the most critical information in the source images to transfer the captured data into a fused image had been solved, with the slightest change, especially distortion or loss. However, they could suffer from decreased contrast [20]. To address this problem, various multi-scale decomposition transforms (MSDT) methods were proposed, e.g., Laplacian pyramid [21] and dual-tree complex wavelet (DTCWT) [22], which decomposed the source images into multi-scale representations with high and low-frequency information. However, because of the different dynamic range of the VIS and IR images, the fused images sometimes have the excessive shift effects of the luminance components, resulting in color distortion in RGB space [23]. Meanwhile, because of the significant differences in the luminance components between the VIS and IR images, the features from the IR images may diminish the fundamental perceptual information of the VIS images.

Edge-preserving smoothing technology was applied to fuse the IR and VIS images to reduce halo artifacts [24,25,26]. A novel decomposition framework based on Gaussian curvature filtering was proposed in [27]. The fusion performance is better than simple coefficient weighting, but the complex weight computing and decomposition could result in high computational cost [28]. In order to reduce calculative cost, the mean filtering was employed in [29] for a two-layer decomposition-based IR and VIS image fusion. The guided-filter-based context enhancement (GFCE) [30] method enhanced the VIS images based on a two-scale decomposition obtained by the guided image filtering (GIF) [31]. The GFCE contains bright and conspicuous targets, but the background of the fused images is distorted due to brightness and enhanced details. Because of the excessive enhancement, artifacts, and distortions appear in the fused images by the GFCE. A new anisotropic diffusion-based image fusion (ADF) [32] filtered each image using the anisotropic diffusion process to extract valuable information from base and detail layers. Anisotropic diffusion filter generates coarser resolution images by using region smoothing to overcome the shortcoming of isotropic diffusion [33]. However, the ADF uses complex edge-preserving filters, which is relatively time-consuming. A Bayesian fusion model was proposed in [34] to measure and handle the uncertainty better so as to maintain the detail information from source images while the computational cost of the algorithm also needs to be reduced. The problems, such as loss of fine details, over-sharpened, color distortion, and degradation of contrast still exist [8,35]. Consequently, the fusion quality of the IR and VIS images requires to be enhanced in terms of contrast, sharpness and detail information. It is thus desired to develop an effective algorithm for the fusion of the IR and VIS images.

**Figure 1:**
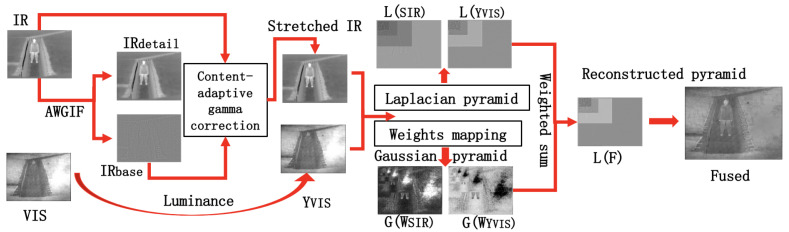
Proposed algorithm.

In this paper, a new multi-scale algorithm is introduced to fuse the IR and VIS images. The proposed algorithm is based on the following two observations: (1) the dynamic ranges of the IR images could be narrower than those of the VIS images in the scenes which are composed of cloud, human subjects, trees, and plants or objects with similar temperature [36]; and (2) bright regions of the IR images usually include more details demanded by the VIS images. The gamma correction can be applied to increase the dynamic range of the IR image. Nevertheless, the gamma correction could magnify noise in smooth regions and the visual quality could be reduced. The adaptive weighted GIF (AWGIF) in [37] is adopted to decompose the IR image into two layers. Only the base layer is stretched by using a content-adaptive gamma correction algorithm. The detail layer is kept to prevent noise from being amplified. The proposed stretch component improves the image quality and reduces the color distortion of the fused image. The fusion is guided by a set of quality measures, such as contrast and exposedness, to extract useful information in both images, which are consolidated into weight matrices [38]. However, unlike the fusion of differently exposed VIS images [39,40], the measures in [38] are not always applicable to the IR images. The contrast measure range of the VIS image is often more expansive than the IR image. The weight matrices of the IR images will be smaller and the fused images tend to the VIS images, losing the useful information of the IR images. In order to better keep the corresponding information of the two images in the weight matrices and assign a high weight to important elements in the stretched IR images, the proposed algorithm performs mapping adjustment on the contrast measure of the stretched IR image. The regions with large intensities should be assigned larger weights than those with small values in the exposedness measure. The parts with higher brightness values in the IR image should be emphasized, such as humans. Therefore, different exposedness parameters are required to adjust the weights of the bright and dark regions. The proposed new weight matrices can strengthen and enrich important information to ensure that the fused image is in line with the human visual system before subsequent analysis and processing. In our experiments, our algorithm is compared with several state-of-the-art fusion algorithms on public datasets. The experiment results show that our algorithm can usually highlight salient objects better and have advantages in grasping the overall luminance contrast and vitality. It is suitable for low-light scenes and situations where the VIS and IR images in the grayspace are quite different, conforming to the human visual sense. The overall scheme of the proposed algorithm is shown in Figure 1. Two main contributions of this paper are: (1) a simple content-adaptive gamma correction algorithm, which stretches the dynamic range of the IR image according to the dynamic range of the VIS image. The algorithm can alleviate the possibly large differences between the dynamic ranges of the IR and VIS images. Subsequently, the excessive shifts of brightness and the color distortion of the fused images are significantly reduced; and (2) novel weight measures for the IR image, which can achieve weight matrices that are more in line with the characteristics. The weight matrices can be used for fusion with IR and panchromatic or RGB VIS images for a wide range of applicability.

The remainder of this paper is organized as follows. Section 2 describes the stretch of the IR images, including the dynamic range of the IR images and the content-adaptive gamma correction by the AWGIF. Section 3 contains the proposed multi-scale fusion of the IR and VIS images. Section 4 illustrates the comparison of the fusion results with some parameters and existing state-of-the-art algorithms on public datasets. Section 5 provides the conclusions.

## 2. Edge-Preserving Stretch of the IR Images

**Figure 2:**
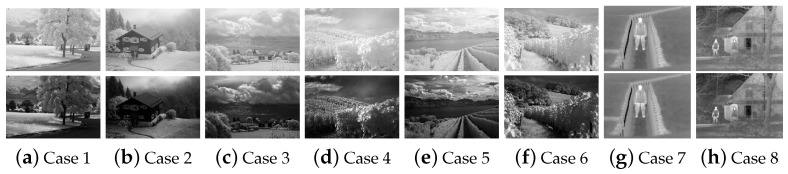
Comparison of dynamic range of the IR images and the stretched IR images. (**a**–**f**) are the scenes which contain more trees and plants. (**g**,**h**) are the other scenes.

Dynamic range of an IR image could be narrower than the corresponding VIS image [36]. For example, trees and plants are highly reflective in the IR image and thus appear much brighter than they do in the VIS image. Cloud is usually white in an IR image. The IR image of a scene that is composed of trees, grass, and clouds has a narrow dynamic range. Eight examples are given in cases 1–8 in the first row of Figure 2. An IR image of a scene which consists of objects with similar temperature also has a narrow dynamic range.

The gamma correction can be applied to stretch the dynamic range of the IR image directly. However, the noise could be amplified in this way. Here, the AWGIF is applied to stretch the IR image because it reduces noise better than the filters in [31,41,42]. Let *Z* be an IR image to be processed and also be the guidance image. *Z* is firstly decomposed into base and detail layers by the AWGIF as:

(1) $$\begin{array}{c} Z=Z_{b}+Z_{d}, \end{array}$$

where the base layer $Z_{b}$ consists of homogeneous regions with sharp edges, and the detail layer $Z_{d}$ is texture or noise.

Since the processed image and the guidance image are the same, $Z_{b}\left(p\right)$ is assumed to be a linear transform of Z(p) in the window $\Omega_{\;r}\left(p^{\prime }\right)$ [31]:

(2) $$Z_{b}\left(p\right)=a_{p^{\prime }}Z\left(p\right)+b_{p^{\prime }}\;\forall p\in \Omega_{\;r}\left(p^{\prime }\right),$$

where $a_{p^{\prime }}$ and $b_{p^{\prime }}$ are two constants in the window $\Omega_{\;r}\left(p^{\prime }\right)$. $\Omega_{\;r}\left(p^{\prime }\right)$ is a square window centered at the pixel $p^{\prime }$ of a radius r.

The $a_{p^{\prime }}$ and $b_{p^{\prime }}$ are obtained by solving the following quadratic optimization problem:

(3) $$\begin{array}{c} \arg\min_{a_{p^{\prime }},b_{p^{\prime }}}\left\{\sum_{p\in \Omega_{\;r}\left(p^{\prime }\right)}\left[\Gamma_{p^{\prime }}^{Z}{\left(Z_{b}\left(p\right)-Z\left(p\right)\right)}^{2}+\lambda a_{p^{\prime }}^{2}\right]\right\}, \end{array}$$

where λ is a regularization parameter which penalizes a large $a_{p^{\prime }}$. The values of *r* and λ for the fusion of panchromatic VIS and IR images are larger than those for the fusion of RGB VIS and IR images.

To reduce the halo artifacts, the edge-aware weighting $\Gamma_{p^{\prime }}^{Z}$ is defined as [41]:

(4) $$\begin{array}{c} \Gamma_{p^{\prime }}^{Z}=\frac{1}{N}\sum_{p=1}^{N}\frac{\sigma_{Z,1}^{2}\left(p^{\prime }\right)+\varepsilon }{\sigma_{Z,1}^{2}\left(p\right)+\varepsilon }, \end{array}$$

$\sigma_{Z,1}^{2}\left(p^{\prime }\right)$ is the variance of *Z* in the window $\Omega_{1}\left(p^{\prime }\right)$. *N* is the total number of pixels in the image *Z*. ε is a small constant and it is 1 for an 8-bit image in the range [0, 255].

The optimal values of $a_{p^{\prime }}$ and $b_{p^{\prime }}$ are computed as:

(5) $$\begin{array}{c} \left\{\begin{array}{c} a_{p^{\prime }}^{*}=\frac{\Gamma_{p^{\prime }}^{Z}\sigma_{Z,\;r}^{2}\left(p^{\prime }\right)}{\Gamma_{p^{\prime }}^{Z}\sigma_{Z,\;r}^{2}\left(p^{\prime }\right)+\lambda }\\ b_{p^{\prime }}^{*}=-\left(a_{p^{\prime }}^{*}-1\right)\mu_{Z,\;r}\left(p^{\prime }\right) \end{array}\right., \end{array}$$

$\mu_{Z,\;r}\left(p^{\prime }\right)$ is the mean values of *Z* in the window $\Omega_{\;r}\left(p^{\prime }\right)$. A weighted averaging method is adopted as:

(6) $$\begin{array}{c} \left\{\begin{array}{c} {\bar{a}}_{p}=\frac{1}{W_{p}^{sum}}\sum_{p^{\prime }\in \Omega_{\;r}\left(p\right)}\left(W_{p^{\prime }}a_{p^{\prime }}^{*}\right)\\ {\bar{b}}_{p}=\frac{1}{W_{p}^{sum}}\sum_{p^{\prime }\in \Omega_{\;r}\left(p\right)}\left(W_{p^{\prime }}b_{p^{\prime }}^{*}\right) \end{array}\right., \end{array}$$

where $W_{p}^{sum}$ is $\sum_{p^{\prime }\in \Omega_{\;r}\left(p\right)}W_{p^{\prime }}$. $W_{p^{\prime }}$ is given as $\exp^{-\eta {\left(a_{p^{\prime }}^{*}-1\right)}^{2}\sigma_{Z,\;r}^{2}\left(p^{\prime }\right)}$. η is a positive constant and it is empirically set to 36 rather than 200 in [42] if not specified. When η is set to a larger value, gradient reversal artifacts will be more obvious, which can be found near the edges. The filtered images also become over-sharpened [42]. The final value of $Z_{b}\left(p\right)$ is given as:

(7) $$Z_{b}\left(p\right)={\bar{a}}_{p}Z\left(p\right)+{\bar{b}}_{p}.$$

The complexity of the AWGIF is O(N) for an image with N pixels which is the same as that of the GIF. The AWGIF can be applied to simultaneously smooth and sharpen an image as in [43].

Following [30,44,45], the dynamic range is regulated on the base layer in the log domain $\tilde{Z_{b}}$ by using a scale factor γ which maps the base layer to a user-controllable base contrast. The magnitude of the detail layer in the log domain $\tilde{Z_{d}}$ is unchanged, therefore, the image details can be preserved. The operation can be expressed as:

(8) $${\tilde{Z}}_{e}\left(p\right)=\gamma \tilde{Z_{b}}\left(p\right)+\tilde{Z_{d}}\left(p\right),$$

where $\tilde{Z_{b}}$ and $\tilde{Z_{d}}$ are the base and detail layers in the log domain, respectively. γ is defined as:

(9) $$\begin{array}{c} \gamma =\max\{\frac{8}{\log_{2}\frac{max(Z_{b})+1}{min(Z_{b})+1}},1\}. \end{array}$$

γ is great than or equal to 1 for the cases 1–6 in Figure 2. It could be almost 1 for IR images with broad dynamic range, such as the cases 7 and 8 in Figure 2. When γ is greater than 1, the dynamic range of the large gray value area becomes enlarged and the contrast would be improved. As shown in the second row of Figure 2, the stretched IR images in eight cases are given. The overall gray value of the image becomes smaller.

The stretched image can transfer from log domain back to real domain as $\exp^{\left({\tilde{Z}}_{e}\left(p\right)\right)}$. Combining the Equation (8), a content-adaptive gamma correction algorithm is proposed to stretch the IR image as:

(10) $$\begin{array}{c} Z_{e}\left(p\right)=Z_{b}^{\gamma }\left(p\right)Z_{d}\left(p\right). \end{array}$$

The IR image can be adaptively stretched by the original input and the base layer. Obviously, only the base layer is stretched while the detail layer is kept. The noise is also prevented from being amplified in the smooth regions.

**Figure 3:**
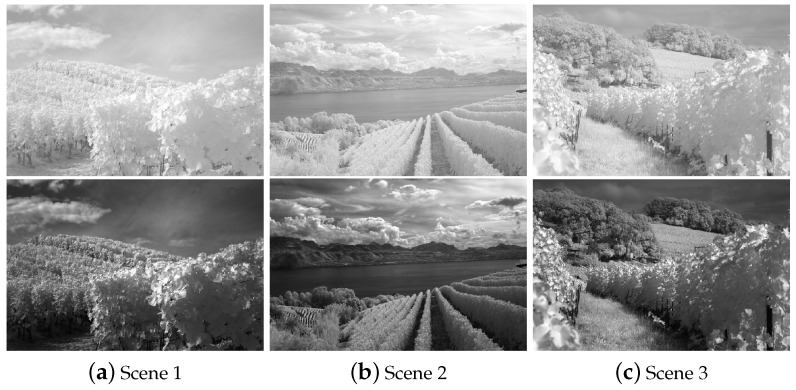
IR images and the effects of γ on the base layer of the stretched IR images.

Figure 3 shows the effects of γ on the base layer in Equation (10). When γ>1, the dynamic range of the large gray value area has broadened while the intensities become smaller.

Although the maximum intensity of $Z_{e}\left(p\right)$ is considered in Equation (10), it is also possible that some intensities of the stretched IR image would be higher than 255 (which can be normalized as 1) and need to be clipped. Therefore, in order to extend the dynamic range and simultaneously keep the output not larger than the maximum intensity, let the normalized maximal and minimal values of $Z_{e}\left(p\right)$ are $Z_{e,M}$ and $Z_{e,m}$, respectively. The output stretched IR image is:

(11) $$\begin{array}{c} {\hat{Z}}_{e}\left(p\right)=\frac{Z_{e}\left(p\right)-Z_{e,m}}{Z_{e,M}-Z_{e,m}}. \end{array}$$

## 3. Multi-Scale Fusion of the IR and VIS Images

The overall scheme of the proposed algorithm is shown in Figure 1, where the input IR and VIS images are linear normalized to (0, 1) before the fusion. To fuse the IR and VIS images, new weight matrices are first proposed according to the characteristics of both images. Then, the stretched IR and VIS images are fused by using the new weight matrices.

### 3.1. Weights of the Stretched IR and VIS Images

In the panchromatic scene, the VIS image is captured as a grayscale image which is single channel and the same as luminance channel *Y*. In the RGB scene, the image is rich in color information. The VIS image is composed of RGB channels, so the luminance channel *Y* is extracted from the VIS image as a grayscale image for further processing. Y(p) can be calculated with the R, G, and B channels as: Y(p)=0.299∗R(p)+0.587∗G(p)+0.114∗B(p).

The proposed weights for the stretched IR and VIS images are defined as:

(12) $$\begin{array}{c} W\left(p\right)={\left(C\left(p\right)\right)}^{w_{c}}*{\left(E\left(p\right)\right)}^{w_{e}}, \end{array}$$

where C(p) and E(p) mean contrast and exposedness measures, respectively. *p* is the pixel in image *I*. $w_{c}$ and $w_{e}$ correspond to the weight exponents, which are set to default value 1 [38]. A Laplacian filter $L_{f}$ is adopted to the grayspace $I_{g}$ of each image I(p). The absolute values of the filter responses are taken to achieve the contrast intensities as:

(13) $$\begin{array}{c} C\left(p\right)=L_{f}⊗I_{g}\left(p\right). \end{array}$$

Here, the operator ⊗ indicates a 2D convolution operation.

**Figure 4:**
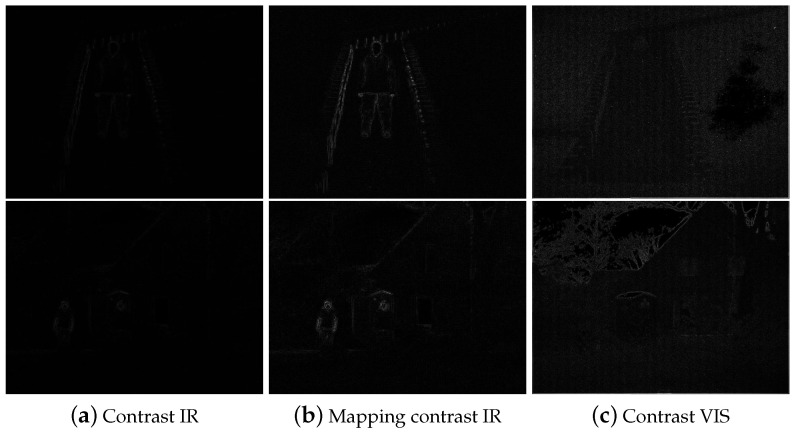
Contrast measure results of the stretched IR and VIS images.

The first column in Figure 4 demonstrates the contrast measure of the stretched IR images of cases 7 and 8 in Figure 2. Additionally, the third column represents the contrast measure of the VIS images. Because the VIS image is high in contrast and texture, the intensity range is greater than the IR image, brightening the display. It tends to assign a high weight to some parts of the VIS image. Through increasing the dynamic range of the IR image, the brightening artifacts have been reduced, alleviating the effect of excessive brightness drift. However, in order to prevent the luminance value of the focus point, such as people, in the IR image from being reduced after fusion, the proportion of this part in the weight matrices should be prominent. Because the influence of each measure is combined by multiplication, the part with higher brightness in the IR image has a lower value in weight matrices, so some parts of the fused image that the IR image focuses on, such as people, become dim. In order to prevent the fused image dominated by the VIS image, the contrast measures of the stretched IR images are mapped to those of VIS images as the following equation so that the two are in the same order of magnitude.

(14) $$\begin{array}{c} {\hat{C}}^{\prime }\left(p\right)=\left(C_{vis_{M}}-C_{vis_{m}}\right)\frac{\hat{C}\left(p\right)-{\hat{C}}_{m}}{{\hat{C}}_{M}-{\hat{C}}_{m}}+C_{vis_{m}}, \end{array}$$

where $C_{vis_{M}}$ and $C_{vis_{m}}$ are maximal and minimal values of the contrast measure of the VIS image $C_{vis}$, respectively. Additionally, ${\hat{C}}_{M}$ and ${\hat{C}}_{m}$ are maximal and minimal values of the contrast measure of the stretched IR image $\hat{C}\left(p\right)$, respectively.

The second column in Figure 4 demonstrates the mapping results of contrast measure of the stretched IR images in Equation (14). It can be shown the intensities increase and the edges are clearer than the first column.

In the exposedness term, each intensity $I_{g}\left(p\right)$ is weighted based on how close it is to 0.5 using a Gauss curve:

(15) $$\begin{array}{c} E\left(p\right)=\left\{\begin{array}{cc} \exp^{\frac{-{\left(I_{g}\left(p\right)-0.5\right)}^{2}}{2*0.2^{2}}} & I_{g}\left(p\right)<0.5\\ \exp^{\frac{-{\left(I_{g}\left(p\right)-0.5\right)}^{2}}{2*\phi^{2}}} & I_{g}\left(p\right)\geq 0.5 \end{array}\right. \end{array}$$

The parameter ϕ is set to 0.2, which is adopted in the VIS images. Instead of using the same value in the stretched IR images, the value is set as 0.375 to improve the weight of the highlighted part in the stretched IR images.

### 3.2. Fusion of the Stretched IR and VIS Images

The Gaussian and Laplacian pyramids are combined to accomplish the fusion [38]. The fused Laplacian pyramid is obtained by combining the Laplacian pyramids at each level of grayscale images and decomposition weighted by corresponding Gaussian pyramids as:

(16) $$\begin{array}{c} L{\left[F(p)\right]}_{l}=G{\left[W_{\hat{Y_{VIS}}}\left(p\right)\right]}_{l}*L{\left[Y_{VIS}\left(p\right)\right]}_{l}\\ +G{\left[W_{\hat{\hat{Z_{e}}}}\left(p\right)\right]}_{l}*L{\left[{\hat{Z}}_{e}\left(p\right)\right]}_{l}, \end{array}$$

where $G{\left[X(p)\right]}_{l}$ and $L{\left[X\left(p\right)\right]}_{l}$ are the $l^{th}$ level of Gaussian pyramid and Laplacian pyramid of image *X* or a matrix *X*, respectively. $Y_{VIS}\left(p\right)$ represents the luminance channel Y extracted by the VIS image. $W_{\hat{X}}$ is the normalized weight between two images.

The fused pyramid $L{\left[F(p)\right]}_{l}$ is then reconstructed to obtain F(p), which is the fused result of the grayspace of the VIS image and stretched IR image. For smooth blending, the pyramid is decomposed to the largest $l^{th}$ power of two that does not exceed the minimum value of the size in images and is up to the level of one pixel. If the VIS image has three channels, after obtaining F(p), all the three channels are adjusted according to the ration $F\left(p\right)/Y_{VIS}\left(p\right)$.

## 4. Experimental Results

The proposed algorithm is compared with ten advanced algorithms, including wavelets [17], MSVD [18], CVT [19], DTCWT [22], GFCE [30], ADF [32], Bayesian [34], multi-level gaussian curvature filtering (MLGCF) [27], weight-map-guided [35], and adaptive fast enhancement [8] to prove its effectiveness. Our experiments are performed using MATLAB Code on a computer with 2.6 GHz 6-Core Intel Core i7 and 16 GB 2400 MHz memory. Datasets and results can be achieved from [46]. Readers are invited to view the electronic version of the paper for a better appreciation of the difference among images.

### 4.1. Performance Evaluation Indices

Measuring the image fusion results by objective fusion evaluation indicators is necessary, which quantitatively simulates the human visual system to perceive the quality of images. Subjective evaluation relies on the naked eyes to make a subjective judgment on the effect of the fused image that is sometimes inaccurate. That is because in human evaluation, many subjective factors affect the evaluation results. Each subject’s intuitive sense will result in erroneous image fusion evaluation. Only one evaluation index will still produce incomplete judgments on evaluating the results. Therefore, multiple indicators are needed to comprehensively assess the results of various fusion algorithms. Three indices, namely, entropy (EN), mutual information (MI), and cross entropy (CE) [47,48,49] are used to evaluate the capability of the fusion results of different algorithms quantitatively. These indices are defined as follows:

The EN reflects the amount of average information contained in the image and represents the aggregation characteristics of the grayscale distribution of the image [16]. The larger the value, the greater the data collected, and the more image details retained. This index is defined as:

(17) $$\begin{array}{c} EN_{\left(F\right)}=-\sum_{l=0}^{255}\frac{F{\left(l\right)}_{Num}}{M*N}log_{2}\frac{F{\left(l\right)}_{Num}}{M*N}\;\left(F{\left(l\right)}_{Num}\neq 0\right), \end{array}$$

where $F{\left(l\right)}_{Num}$ is the number of the pixels with the gray level *l* in the image *F*. *M* and *N* represent the length and width of the image *F*, respectively.

The MI can measure the degree of similarity between two images, that is, how much information the fused image has acquired from the original images. Large MI implies the fused image holds more information from the original images. The index is expressed as:

(18) $$\begin{array}{c} MI_{\left(fused,vis,ir\right)}=MI_{\left(fused,vis\right)}+MI_{\left(fused,ir\right)}, \end{array}$$

where $MI_{\left(fused,vis\right)}$ and $MI_{\left(fused,ir\right)}$ represent the amount of information in the VIS and IR images contained in the fused images, respectively. The MI is a particular case of a wide relative EN, which can be calculated as follows:

(19) $$\begin{array}{c} MI_{\left(A,B\right)}=EN_{\left(A\right)}+EN_{\left(B\right)}-U_{\left(A,B\right)}, \end{array}$$

where $U_{\left(A,B\right)}$ is defined as follows:

(20) $$\begin{array}{c} U_{\left(A,B\right)}=\sum_{a=0}^{255}\sum_{b=0}^{255}\frac{AB{\left(a,b\right)}_{Num}}{M*N}log_{2}\frac{AB{\left(a,b\right)}_{Num}}{M*N}\\ (AB{\left(a,b\right)}_{Num}\neq 0), \end{array}$$

where $AB{\left(a,b\right)}_{Num}$ represents the number of pixels in the same position in image *A* and image *B*, which the gray level in image *A* is *a*, and in image *B* is *b*.

The smaller the CE value, the better effect is. The more miniature the value, the closer it is to the original images, and the more details are collected. CE can be expressed as follows:

(21) $$\begin{array}{c} CE_{\left(fused,vis,ir\right)}=\sqrt{\frac{CE_{\left(fused,vis\right)}^{2}+CE_{\left(fused,ir\right)}^{2}}{2}}, \end{array}$$

where $CE_{\left(fused,vis\right)}$ and $CE_{\left(fused,ir\right)}$ represent the probability distributions of information in the VIS and IR images contained in the fused images, respectively, which are given as:

(22) $$\begin{array}{c} CE_{\left(A,B\right)}=\sum_{l=0}^{255}\frac{B{\left(l\right)}_{Num}}{M*N}log_{2}\frac{A{\left(l\right)}_{Num}}{B{\left(l\right)}_{Num}}\\ (A{\left(l\right)}_{Num},\;B{\left(l\right)}_{Num}\neq 0), \end{array}$$

where $A{\left(l\right)}_{Num}$, $B{\left(l\right)}_{Num}$ represents the same sense with $F{\left(l\right)}_{Num}$.

### 4.2. Selection of Parameters

The parameter settings affect the performance of the proposed algorithm. The proposed algorithm mainly contains two parameters, namely, *r* and λ. *r* is the radius of the window $\Omega_{\;r}\left(p^{\prime }\right)$, which determines the degree of information extraction and affects the efficiency of edge-preserving smoothing. λ in Equation (3), which penalizes $a_{p^{\prime }}$ and prevents the value of $a_{p^{\prime }}$ from being too large.

Our goal is to obtain suitable parameters so that the images can obtain better results on human visual effects and evaluation indices. The two parameters usually have turning points. The evaluation indices improve if the value does not reach the turning point. Conversely, beyond the turning point, the evaluation effect begins to decline. If *r* is small, the filter window is small, so the weighting coefficient of the edge-aware is too fine. Therefore, the sharpening effects strengthen and some unnecessary details will be extracted, which also causes waste of computing resources. On the other hand, if the filter window is too large, it enhances the smoothing effect, and some texture information lost, resulting in a decline in image quality. If λ is too small, the degree of punishment will be reduced, and the impact on $a_{p^{\prime }}$ will be weakened. Correspondingly, too large λ leads to a small $a_{p^{\prime }}$, which makes it difficult for the two layers to extract sufficient edge-preserving information from the original image. The datasets are derived from the aligned VIS and IR image pairs in TNO Human Factors, which contain various images taken in different military-related scenes [16]. We quantitatively compare sequences, namely, nato-camp, which includes 32 image pairs to choose proper parameters.

**Figure 5:**
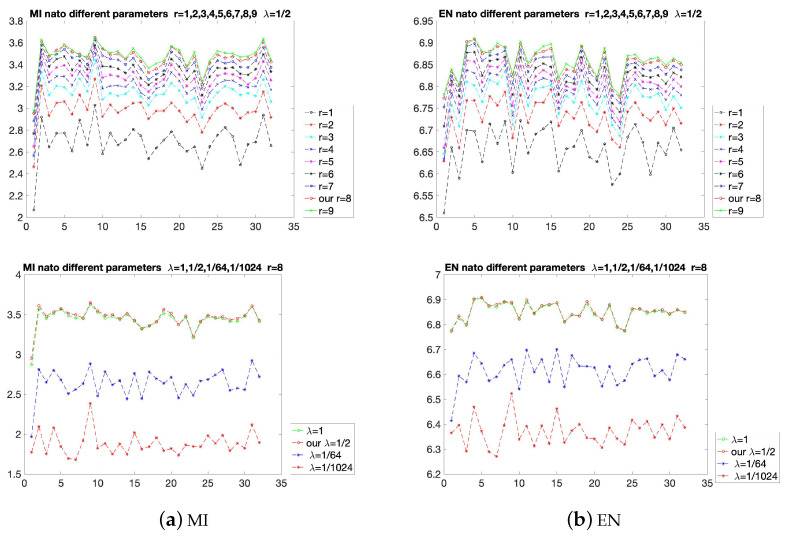
Quantitative comparison of the two indicators, MI and EN on the nato-camp sequences. Some different parameters are used for comparison.

The results of the proposed method with different parameters are shown in Figure 5. The MI and EN are used to evaluate the parameters. The EN considers the statistical characteristics based on the unreferenced images, and the MI is used to measure the integrated information from the referenced source images. The choices of *r* are shown in the first row of Figure 5. It can be seen that when λ is fixed, adjusting the value of *r* will affect the evaluation indicators. As *r* increases from 1 to 8, although the increasing interval may be different, the overall value of the curve increases. However, when *r* is increased from 8 to 9, the values of some of the 32 points decrease, resulting in some turning points. Therefore, to avoid reducing the fusion effects at certain points, the value of *r* should be selected as 8. In the second row of Figure 5, *r* is fixed as 8, then λ is increased from $\frac{1}{1024}$ to 1. Similar to the result of *r*, some turning points appear when λ is equal to 1. So the value of λ is selected as $\frac{1}{2}$. In the experiments of comparison with state-of-the-art methods, λ is fixed at $\frac{1}{2}$ and *r* is fixed at 8, which have excellent visual effects in most fusion results.

**Figure 6:**
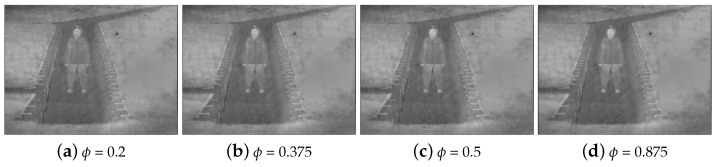
Comparison of different exposedness parameter ϕ.

ϕ in Equation (15) is another adjustive parameter. With the increase in this parameter, the value in the exposedness measure of the point with the grayscale intensity greater than 0.5, such as 1, in the stretched IR image will rise, but it will not exceed 1. The brightness of the fused image in this part is improved. The effects of the fused images when ϕ takes different values are represents in Figure 6. Compared with (a), (b), (c), and (d) in Figure 6, it can be shown that when ϕ is larger than 0.375, which has little impact on brightness improvement. If it is too large, it will cause excessive brightness shifts in some scenarios, loss of details, and affect the quality of the image. The details are explained in the fifth subsection. Considering the above reasons, it is chosen as 0.375 as a compromise parameter.

### 4.3. Comparison with State-of-the-Art Methods

The nato-camp sequences are first selected to compare the proposed algorithm with different state-of-the-art methods. An image fusion method should focus more on the relationship between the fused image and the original images. Therefore, in order to evaluate various fusion methods more comprehensively, the CE is added in the comparison with different methods.

**Figure 7:**
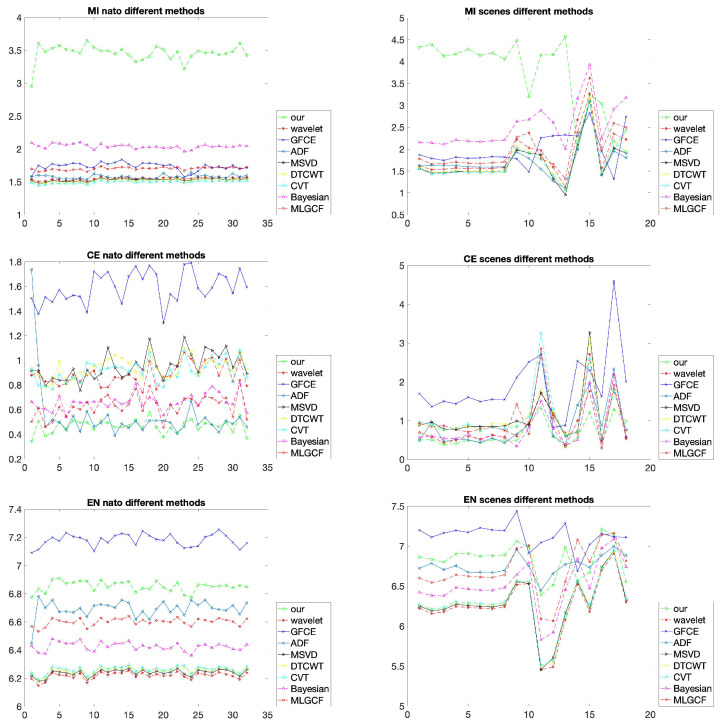
Comparison of the three indicators, MI, CE, and EN on the nato-camp sequences and representative scenes. Different methods are used for comparison.

The average running times of different fusion methods on the nato-camp sequences are provided in Table 1. It can be shown that the proposed method is faster than other methods except the DTCWT. The proposed method outperforms other methods from the MI and CE points of view on the nato-camp sequences, as shown in the left half of Figure 7. In terms of the MI, the proposed method approximately achieves twice higher scores of all the other methods. Except for the ADF, Bayesian and MLGCF, the values of other methods are approximately bigger than twice the proposed method under the CE index. The index EN can directly evaluate the quality of the resultant image without relying on the original images. It can be shown that the GFCE method achieves the highest scores and our method is second. This is because the VIS images are modified to enhance the luminance before the fusion in the GFCE. The average objective values of different methods in the nato-camo sequences are demonstrated in Table 2.

Subsequently, some images in various representative scenarios that contain informative and high contrast regions, such as building, plant, soldier in the grass, soldier in a trench, are tested to prove the proposed method’s general applicability. All the methods are compared quantitatively on 18 different representative image pairs. Four typical scenes are chosen to compare them qualitatively.

The average running times of 18 image pairs for all methods are listed in Table 3. The results show that our method and the DTCWT are considerably faster than all other competitors. In the execution time of the nato-camp dataset, as shown in Table 1, because the size of the image is small, some methods take more than twice the execution time of our method. When the size of the images becomes larger in other scenes, the gap becomes more pronounced in Table 3. Execution time is crucial in some applications, such as military operations and robot path planning. The average running time of our method is approximately 0.1116 s, which is suitable for real-time fusion tasks.

Similar to the result of the nato-camp sequences, the evaluation indicators of different scenes are shown in the right half of Figure 7. Our method is still very effective in the MI and CE. The more critical aspect of the fusion method should consider the relationship between the fused images and the original images. Although the EN of the proposed method is not the largest, the comparison results still show that our method has a strong correlation with the original images and is in line with the human visual perception. Table 4 shows the average results of scenes in Figure 7.

**Figure 8:**
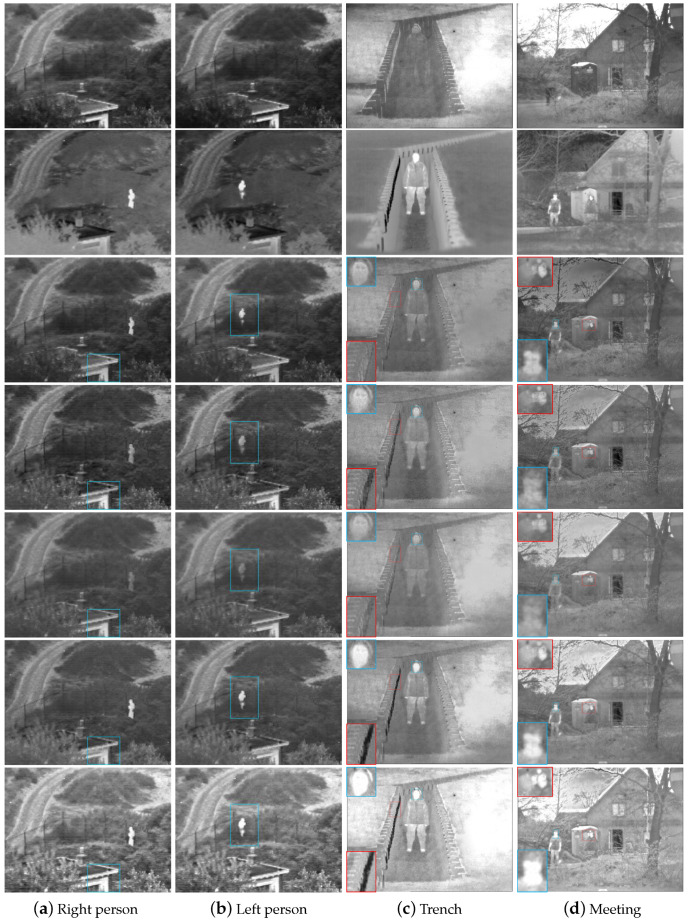
Fusion results on four typical panchromatic VIS and IR images. From top to bottom: the VIS images, the IR images, the fused images of our method, ADF [32], Bayesian [34], MLGCF [27], and GFCE [30].

**Figure 9:**
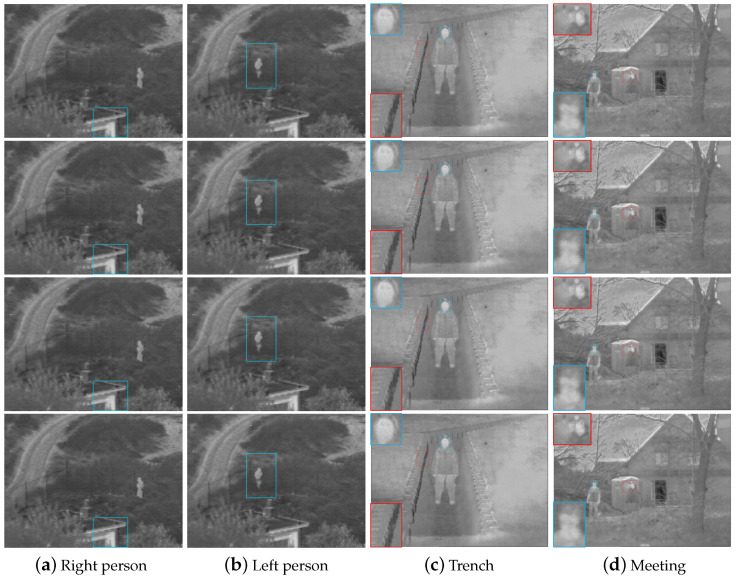
Fusion results on four typical panchromatic VIS and IR images. From top to bottom: Wavelets [17], MSVD [18], CVT [19], and DTCWT [22].

Four examples of image pairs are shown in the first and second rows of Figure 8. The results obtained from the proposed method are shown in the third row of Figure 8. The fourth, fifth, sixth, and seventh rows of Figure 8 represent the results of ADF, Bayesian, MLGCF, and GFCE methods, respectively. It can be shown that the proposed algorithm is able to combine features of the VIS and IR images while preserving important perceptual contents of the backgrounds and details. This can be more clearly evidenced from the fusion results as shown in the third row by comparing them with the VIS and IR images. Due to the lower dynamic range of the IR images, direct fusion with the VIS images would not thoroughly combine the features of high-contrast regions in the VIS image, especially if the corresponding areas in the image have helpful information. Therefore, a content-adaptive gamma correction of the IR image prior to the fusion is reasonably necessary and a noticeable context enhancement can be obtained for images. Other state-of-the-art fusion methods are compared with the proposed method in Figure 9. The first and second columns show that the scenery and the man with our method are more clear than other methods.

Except for the MLGCF and GFCE methods, the people in the fusion results of other methods are not prominent and can hardly be distinguished from the backgrounds in the first and second columns of Figure 8 and Figure 9. The fine details of the eaves and handrails are preserved better by the proposed method as shown in blue rectangles, which fully expresses the contrast information. Our proposed method, the MLGCF and GFCE methods highlight the target areas, which are conducive to subsequent tracking and detection. However, our proposed method preserves edge better and increases more dynamic range than the GFCE method, which is suitable for the human visual system. On the other hand, the GFCE adopts four-level decomposition while our method only decomposes once. Although the people can be displayed in all four images, the global luminance of the GFCE method is excessively enhanced that it is not comfortable for unaided eyes. The MLGCF method takes more than 10 times the execution time of our method, which is not friendly to real-time tasks. In the third column, the MLGCF and GFCE methods have almost no face details, as shown in blue rectangles. The Bayesian method has low brightness, and the backgrounds of the wavelets, MSVD, CVT, and DTCWT methods are not clear enough. In addition, from the third column of Figure 8 and Figure 9, all contrastive methods tend to produce more or less unnatural “shadow” artifacts in the trench, as shown in red rectangles, mainly due to the low global contrast of the IR images and noise. As for the fusion results listed in the fourth column of Figure 8 and Figure 9, the edge contours of the head in the MLGCF, GFCE, wavelets, MSVD, CVT, and DTCWT methods are not obvious enough, as shown in blue amplified boxes. The boundaries of the low building, the coat and the man are less clear in the ADF and Bayesian methods, as shown in red boxes. Overall, the proposed method highlights the people and considers global contrast and details. It can produce better fusion results for vision and further enhance the dynamic range of the whole scene.

### 4.4. Fusion of RGB VIS and IR Images

As mentioned in the second section, the dynamic range of the IR image in the scene which contains plants is usually narrow [36]. The VIS and IR images are merged in the grayspace, and then a ratio is obtained by dividing it with the luminance channel *Y* of the VIS image if needed. The RGB space values of the VIS image are multiplied by this ratio to obtain the final results, which will cause the color of the fused image to be brighter and distorted. This situation is pronounced for the scenes with plants and grasses.

**Figure 10:**
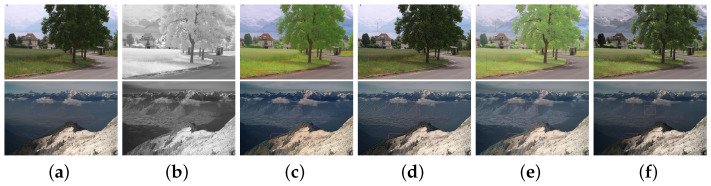
Fusion results for RGB VIS and IR images. (**a**) VIS. (**b**) IR. (**c**) Fusion by [35]. (**d**) Fusion by [8]. (**e**) Unstretched. (**f**) Stretched.

The first two columns in Figure 10 are two RGB VIS and IR images sets [50]. The third column includes the fused images by [35]. The results of an adaptive, fast, and non-iterative fusion approach [8] are shown in the fourth column. The fifth column demonstrates the results of the IR images that are not stretched, and the rest of the processes are the same as the proposed algorithm. The last column represents the fusion of the stretched gamma correction IR and VIS images. In the first row, the colors of the trees and grass are distorted in the third and fifth columns. The colors of trees and grass are too bright. In the second row, the green forest in the close range and the valley in the distance appear unnatural colors, as shown in red boxes of Figure 10c,e. The color of the forest is distorted to light green and the far away valleys are distorted by whitening effects. Figure 10d looks sharper than those produced by Figure 10e,f. However, it is over-sharpened and causes color distortion, e.g., hills in the first and second rows and sky in the second row. Using the stretched IR and VIS images for fusion, the color is preserved well. The details of the two images are well complemented, making the image more vivid and comfortable to the human eyes. Clearly, the propose stretch algorithm significantly improves the visual quality of the fused images.

**Figure 11:**
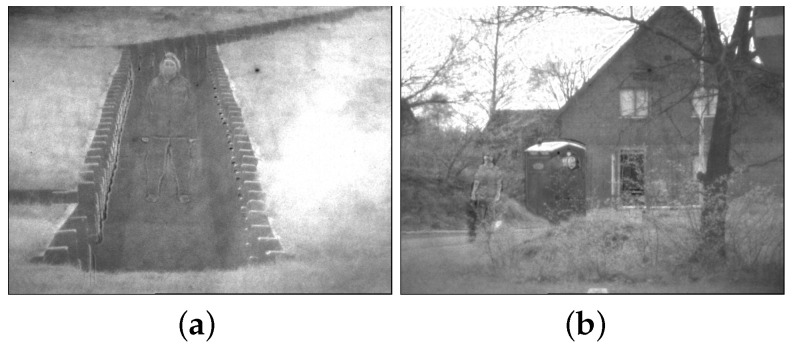
Results by [8] for the fusion of panchromatic VIS and IR images. (**a**) Fusion by [8] in Trench. (**b**) Fusion by [8] in Meeting.

It is worth noting that although the fusion result of [8] is acceptable in the fusion of RGB VIS and IR images, the results of the method in [8] are poor in the fusion of panchromatic VIS and IR images, as shown in Figure 11. The proposed algorithm achieves better results in all scenes,which validates general applicability of the proposed algorithm.

### 4.5. Limitation of the Proposed Algorithm

**Figure 12:**
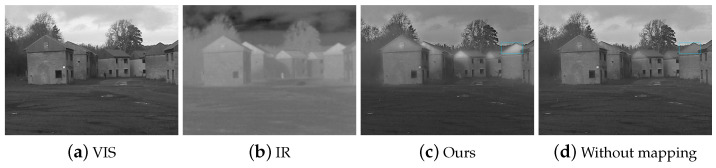
The roof of the building in the IR image is bright and in the VIS image is textured.

ϕ in Equation (15) decides the proportion of the intensity greater than 0.5 in the weight matrices in the image. A larger ϕ will make this effect more significant, thereby making the quality of the image even more degraded. For instance, as shown in Figure 12, the roof of the house is ribbed, and the gray level in the IR image is higher than that in the VIS image. Details of the VIS image are missed in the fused image. Fortunately, the problem could be overcome by designing an adaptive algorithm which will switch on the mapping according to different scenes. As shown in Figure 12d, the details of the VIS image are preserved better by disabling the mapping. In the future, we will develop such an adaptive algorithm.

## 5. Conclusions Remarks

In this paper, we propose a fusion algorithm of the infrared (IR) and visible (VIS) images. The IR image is first stretched using a novel content-adaptive gamma correction to extend the dynamic range if necessary. New weight matrices via contrast and exposedness measures are then introduced by considering the characteristics of the stretched IR and VIS images. Finally, the Gaussian and Laplacian pyramids are combined according to the weight matrices to produce the fused image. The experimental results compare the proposed algorithm with 10 different state-of-the-art fusion algorithms to verify the efficiency. The proposed algorithm achieves better results in fusing both panchromatic and RGB VIS images with IR images, which demonstrates the proposed method’s general applicability. As indicated in [51], a proper detail enhancement algorithm can be applied to improve the fused image subjectively and objectively. The detail enhancement component in [52] will be extended for the fusion of the IR and VIS images. One interesting problem is to determine the value of η by using the quality indices. Another interesting problem is to study the fusion of the IR and VIS images via deep learning based methods. These problems will be studied in our future research. 

## Figures and Tables

**Table 1:** Running times of nato with different methods.

	Ours	Wavelets	GFCE	ADF	MSVD	DTCWT	CVT	Bayesian	MLGCF
Time	***2.3639***	4.3822	5.6738	4.2315	3.2449	**1.3854**	6.7786	5.2462	14.2767

**Table 2:** Objective values of nato With different methods.

	Ours	Wavelets	GFCE	ADF	MSVD	DTCWT	CVT	Bayesian	MLGCF
MI	**3.4579**	1.5512	1.7342	1.5782	1.5292	1.5213	1.4947	***2.0398***	1.7019
CE	**0.4657**	0.8837	1.5930	***0.5453***	0.9521	0.9413	0.9119	0.6647	0.6290
EN	***6.8528***	6.2181	**7.1797**	6.6929	6.2390	6.2535	6.2611	6.4260	6.6009

**Table 3:** Running times of scenes with different methods.

	Ours	Wavelets	GFCE	ADF	MSVD	DTCWT	CVT	Bayesian	MLGCF
Time	***3.2151***	3.2838	10.8168	5.8897	5.7947	**2.1401**	9.5064	9.9251	37.1023

**Table 4:** Objective values of scenes with different methods.

	Ours	Wavelets	GFCE	ADF	MSVD	DTCWT	CVT	Bayesian	MLGCF
MI	**3.7220**	1.8467	1.9845	1.7093	1.6970	1.7371	1.7068	***2.5319***	2.0197
CE	**0.6601**	1.0117	1.9040	0.9279	1.0725	1.0751	1.1186	***0.7838***	0.9088
EN	***6.8365***	6.2451	**7.1315**	6.7550	6.2801	6.2893	6.2979	6.5153	6.6976

## Data Availability

Not applicable.
